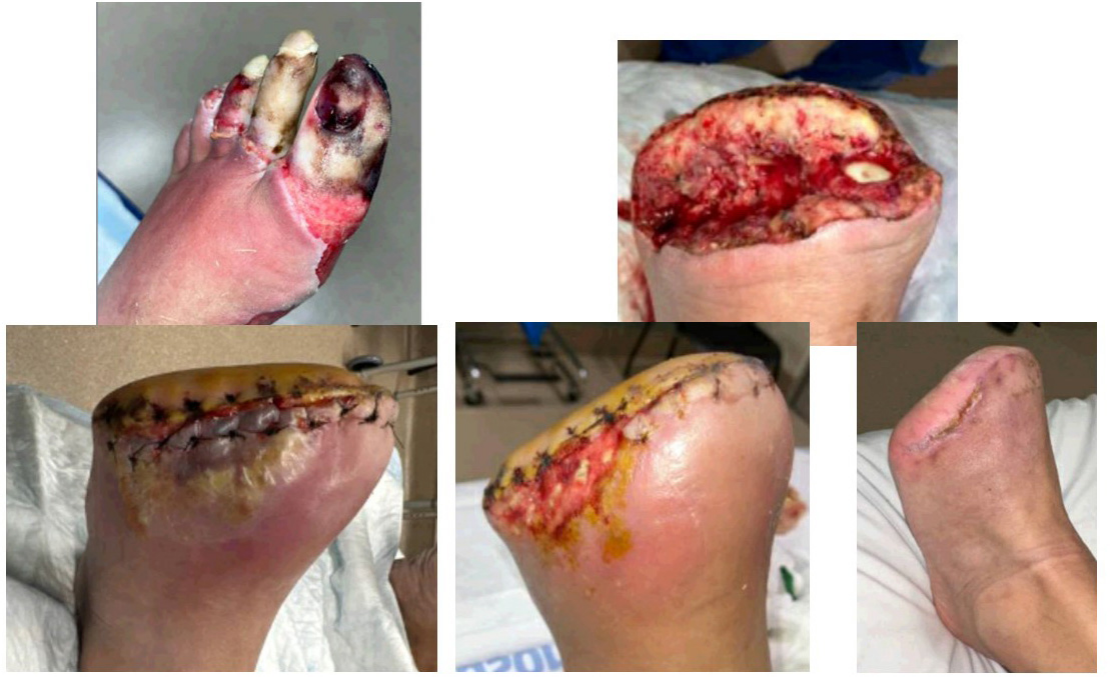# 1000 Hyperbaric Oxygen Therapy for Compromised Residual Limbs

**DOI:** 10.1093/jbcr/iraf019.531

**Published:** 2025-04-01

**Authors:** Nicole Kopari, Robin Draper

**Affiliations:** University of California San Francisco, Fresno, Leon S. Peters Burn Center; University of California San Francisco Fresno

## Abstract

**Introduction:**

With impaired microcirculatory circulation and peripheral neuropathy, diabetics are at an increased risk of developing scald burn injuries and are less likely to heal them. In the setting of impaired microcirculation and a compromised wound bed, hyperbaric oxygen (HBO) therapy provides a potential means to improve the oxygen concentration in the healing wound and may allow a wound to heal that would otherwise require amputation. We present an interesting case using HBO as a salvage option for a compromised residual limb after a diabetic patient underwent a transmetatarsal amputation (TMA) for a scald burn.

**Methods:**

A retrospective chart review was performed on a 64-year-old male with a history of diabetes and peripheral neuropathy who presented to the emergency department (EM) after sustaining a scald burns to his left foot. Patient suffered a burn from his sauna when exposed to a disconnected hose for 10 minutes. He presented with fully demarcated tissue necrosis to left forefoot.

**Results:**

Patient was taken to the operating room (OR) for guillotine TMA secondary to concerns of gangrene in the setting of poorly controlled diabetes. He was placed in wet to dry dressings and 3 days later he underwent formalization of the TMA. Patient was discharged on post-operative day 3 with instructions to remain non-weightbearing and elevate for edema management. The team failed to notice early signs of tissue compromise at his discharge. At his first clinic visit, 10 days after his TMA, concerns for non-compliance with non-weightbearing status, edema, and tissue compromise were identified. The patient was re-admitted to the hospital for wound care, edema management, and consultation with the HBO team for treatment. He underwent 5 sessions (1x/day) of HBO therapy (one 60-minute session and four 90-minute sessions) while inpatient. He had significant improvement after the initial treatment but remained hospitalized until outpatient HBO insurance authorization was obtained. Patient underwent an additional 15 sessions of outpatient HBO (3x/week for a total of 5 weeks) with improvement in the compromised limb decreasing the need for additional surgical revision and eventual wound closure.

**Conclusions:**

This case illustrates HBO therapy as a salvageable option for compromised residual limbs in preventing further need for proximal amputations. Early recognition of ischemic/nonviable tissue is key for providing prompt interventions, including the use of HBO therapy which has been proven to minimize edema, protect the microvasculture, and enhancing host defenses to ward off infection. HBO should be in the algorithm for treatment for compromised residual limbs allowing this patient to forgo a higher level of amputation and minimizing the need for long-term prosthesis.

**Applicability of Research to Practice:**

HBO should be in the algorithm for treatment for compromised residual limbs which may prevent higher level of amputation and minimizing the need for long-term prosthesis.

**Funding for the Study:**

N/A